# The efficacy and safety of sulindac for colorectal polyps: A protocol for systematic review and meta-analysis

**DOI:** 10.1097/MD.0000000000022402

**Published:** 2020-10-09

**Authors:** Qing Long, Liang Ao, Kuo Li, Yan Li

**Affiliations:** a Department of Traditional Chinese Medicine, The Affiliated Hospital of Southwest Medical University; b Department of Orthopedics; c Department of Oncology; d Department of Dermatology, Traditional Chinese Medicine Hospital Affiliated to Southwest Medical University, Luzhou, Sichuan Province, China.

**Keywords:** colorectal polyp, meta-analysis, protocol, sulindac, systematic review

## Abstract

**Background:**

Sulindac has been used for treating colorectal polyps widely. However, the efficacy and safety of sulindac for colorectal polyps are unclear. This study aims to evaluate the efficacy and safety of sulindac for colorectal polyps.

**Methods:**

Randomized controlled trials of sulindac in the treatment of colorectal polyps will be searched in PubMed, EMbase, Cochrane Library, Web of Science, China National Knowledge Infrastructure (CNKI), WanFang, the Chongqing VIP Chinese Science, and Technology Periodical Database, and China biomedical literature database (CBM) from inception to August, 2020. And Baidu Scholar, Google Scholar, International Clinical Trials Registry Platform, and Chinese Clinical Trials Registry will be searched to obtain more relevant studies comprehensively. Two researchers will perform data extraction and risk of bias assessment independently. Statistical analysis will be conducted in RevMan 5.3.

**Results:**

This study will summarize the present evidence by exploring the efficacy and safety of sulindac in the treatment of colorectal polyps.

**Conclusion:**

The findings of the study will provide helpful evidence for the efficacy and safety of sulindac in the treatment of colorectal polyps, facilitating clinical practice and further scientific studies.

**Ethics and dissemination:**

The private information from individuals will not publish. This systematic review also will not involve endangering participant rights. Ethical approval is not required. The results may be published in a peer-reviewed journal or disseminated in relevant conferences.

**OSF Registration number:**

DOI 10.17605/OSF.IO/N5GDH

## 1 Introduction

The colorectal polyp is a mass protruded from the surface of the colorectal. It may be an adenoma or a hypertrophy of the intestinal mucosa, collectively known as a polyp until its pathological nature is determined. Polyps are solitary or multiple, and hereditary or nonhereditary.^[1]^ In recent years, with the improvement of living standard and the lifestyle, the incidence of colorectal polyp shows an obvious rising trend.^[2]^ It may be associated with a high-fat, high-protein diet and lack of physical activity. A number of studies have also shown that metabolic syndrome-related indicators such as blood lipid, blood glucose, and body mass index are correlated with colorectal polyps.^[3,4]^

The formation mechanism of colorectal polyps is complex and affected by a variety of factors. Over a long period of time, various pathogenic factors may act independently or synergistically at different stages, eventually causing cell canceration. Some polyps can develop into colorectal cancer, and colorectal cancer is the third most common cancer worldwide and the second leading cause of cancer-related deaths. Early detection and treatment are the key to improve prognosis. Resection or chemotherapy of colorectal polyps is particularly important.^[5]^ Depending on the severity of symptoms, polyps can be treated with Chinese and Western medicine, colonoscopy, laser, freezing, ligation, and transabdominal or transanal methods.^[6]^ A number of current epidemiological and laboratory studies have shown that nonsteroidal anti-inflammatory drugs (NSAIDs) inhibit or even reverse precancerous lesions in colorectal cancer, and some prospective studies have shown that sulindac reduces the number and size of colorectal polyps in patients with colorectal cancer, especially familial adenomatous polyposis (FAP).^[7,8]^ However, there is no systematic review and meta-analysis regarding the efficacy and safety of sulindac for colorectal polyps. Thus, this study will assess the efficacy and safety of sulindac for colorectal polyps.

## 2 Methods

### 2.1 Study registration

This protocol of systematic review and meta-analysis has been drafted under the guidance of the preferred reporting items for systematic reviews and meta-analyses protocols (PRISMA-P). Moreover, it has been registered on open science framework (OSF) on August 26, 2020. (Registration number: DOI 10.17605/OSF.IO/N5GDH)

### 2.2 Ethics

Ethical approval is not required as there is no patient recruitment and personal information collection, and the data included in our study are from published literature.

### 2.3 Inclusion criteria for study selection

#### 2.3.1 Type of studies

Randomized controlled trials (RCTs) including sulindac for colorectal polyps will be included. The language will be limited to Chinese and English.

#### 2.3.2 Type of participants

All the included cases conform to the “guidelines for management of colorectal polyps,”^[9]^ regardless of nationality, race, age, sex, and source of cases.

#### 2.3.3 Type of interventions

The control group was treated with placebo or no medication; the treatment group was treated with sulindac. The duration of treatment in both groups was not limited.

#### 2.3.4 Type of outcome measures

The main outcome measures were clinical efficacy and polyp disappearance rate. Invalid: the patient's condition was not improved or even aggravated. Effective: the clinical symptoms and signs such as diarrhea or increased frequency of defecation, abdominal tenderness, and other clinical symptoms and signs were alleviated, and the scores of clinical indicators were improved by 50% ∼ 79%; Significant effect: the clinical symptoms and signs such as diarrhea or defecation frequency increased, abdominal tenderness and other clinical symptoms and signs were significantly reduced, and the scores of clinical indicators were improved by >80%. Total effective = significant effective + effective.

Secondary outcome measures were adenoma diameter change and adverse reactions. ① Polyp characteristics: the pathological type of polyps, the number and size of colorectal polyps, the degree of atypical hyperplasia of colorectal polyps. According to the World Health Organization (WHO) classification, the degree of atypical hyperplasia is divided into mild and severe. The adverse pathological features were defined as intraepithelial neoplasia with a diameter of >1 cm or a high degree of atypia. ② Drug safety and adverse reactions: the adverse drug reactions of sulindac in the study group were monitored by outpatient review and emergency telephone emergency system every 6 months.

### 2.4 Exclusion criteria

1. The treatment group used other drugs, such as aspirin, ibuprofen, naproxen, and so on;
2. The outcome indicators of the original study did not meet the requirements;
3. As for duplicate published literature, select the literature with the most complete data;
4. Literature with incorrect or incomplete research data that cannot be obtained after contacting the author.

### 2.5 Search strategy

PubMed, EMbase, Cochrane Library, Web of Science, China National Knowledge Infrastructure (CNKI), WanFang, the Chongqing VIP Chinese Science and Technology Periodical Database, and China biomedical literature database (CBM) were searched by computer to collect RCTs of sulindac for colorectal polyps, and the retrieval time was from the establishment of each database to August 2020. At the same time, search Baidu, Google Scholar, International Clinical Trials Registry Platform (ICTRP), and Chinese Clinical Trials Registry (ChiCTR) to get more comprehensive data. Keywords were “intestinal polyps,” “colorectal polyps,” “familial adenomatous polyposis,” “sporadic colorectal polyps,” “sulindac,” and so on. PubMed retrieval strategies are shown in Table 1.

**Table 1:**
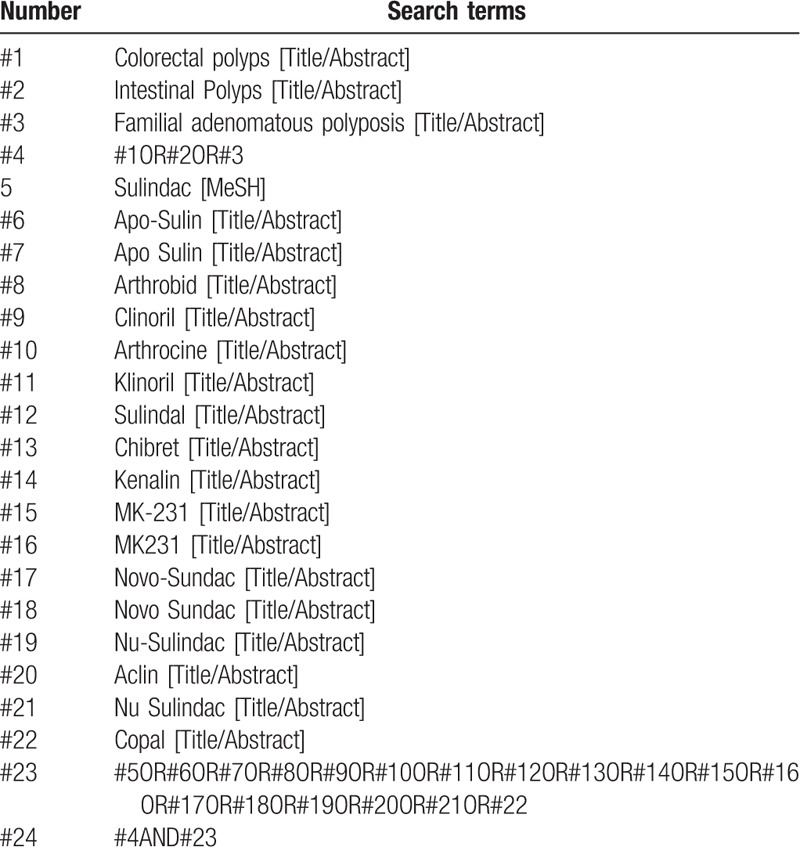
Search strategy in PubMed database.

### 2.6 Data extraction

Endnote X7 was used for literature management. Two researchers independently screened the literature, extracted the data, and cross-checked them. In case of disagreement, the third researcher was consulted to assist in judgment, and the lack of data was contacted with the author as much as possible to supplement. In the process of literature screening, first read the title and abstract, and then read the full text after excluding the obviously unrelated literature to determine whether it is included. Excel 2019 was used to set up a data extraction table to extract data.

**Figure 1:**
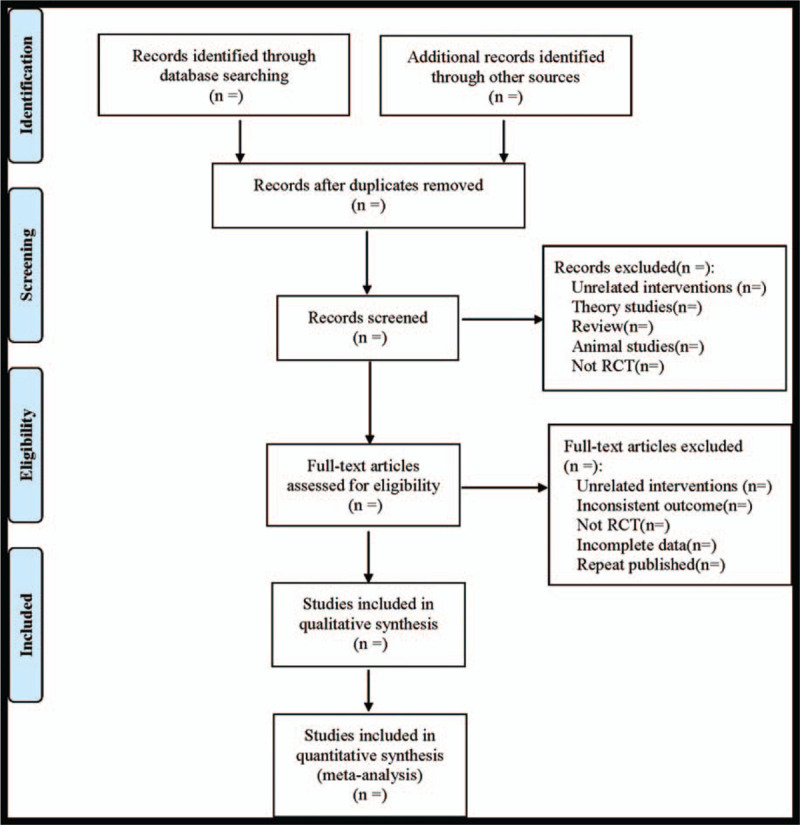
Flow diagram.

The extraction contents were as follows: ① included basic research information (study title, first author, publication time, sample size, sex ratio, average age, and so on); ② information about intervention measures (sulindac used in the treatment group, its dose, course of treatment, and so on); ③ risk evaluation items of bias in RCTs; ④ Related outcome indicators. The literature screening process is shown in Figure 1.

### 2.7 Risk of bias assessment

Two researchers independently evaluated the risk of bias in RCTs in accordance with the Cochrane Handbook of Systematic Reviewers, including the following items: random sequence generation, allocation concealment, blinding of participants and personnel, blinding of outcome assessment, incomplete outcome data, selective reporting, and other bias. The quality of studies was classified as being at of high, unclear, or low risk of bias. In case of disagreement, a third researcher decided.

### 2.8 Statistical analysis

#### 2.8.1 Data synthesis

The RevMan 5.3 software provided by the Cochrane Collaboration was used for statistical analysis. ① For dichotomous variables, relative risk (RR) was used for statistics. For continuous variables, weighted mean difference was selected when the tools and units of measurement indicators are the same, and standardized mean difference was selected with different tools or units of measurement, and all the above were represented by effect value and 95% confidence interval. ② Heterogeneity test: *Q* test was used to qualitatively determine inter-study heterogeneity. If *P* ≥ .1, there was no inter-study heterogeneity, If *P* < .1, it indicated inter-study heterogeneity. At the same time, *I*^2^ value was used to quantitatively evaluate the inter-study heterogeneity. If *I*^*2*^ ≤50%, the heterogeneity was considered to be good, and the fixed-effect model was adopted. If *I*^*2*^ > 50%, it was considered to have significant heterogeneity, the source of heterogeneity would be explored through subgroup analysis or sensitivity analysis. If there was no obvious clinical or methodological heterogeneity, it would be considered as statistical heterogeneity, and the random-effect model would be used for analysis. Descriptive analysis was used if there was significant clinical heterogeneity between the 2 groups and subgroup analysis was not available.

#### 2.8.2 Dealing with missing data

If data are missing or incomplete, we will contact the relevant authors to obtain the data. If not, this study will be removed.

#### 2.8.3 Heterogeneity and subgroup analysis

To reduce the clinical heterogeneity between studies, subgroup analysis was conducted according to the age, which was divided into minors, adults, and the elderly. Subgroup analysis was carried out for the included studies according to the types of colorectal polyps.

#### 2.8.4 Sensitivity analysis

To test the stability of meta-analysis results of indicators, a one-by-one elimination method will be adopted for sensitivity analysis.

#### 2.8.5 Reporting bias

For the major outcome indicators, if the included study was ≥10, funnel plot was used to qualitatively detect publication bias. Egger and Begg test are used to quantitatively assess potential publication bias.

#### 2.8.6 Evidence quality evaluation

The Grading of Recommendations Assessment, Development, and Evaluation (GRADE) will be used to assess the quality of evidence. It contains 5 domains (bias risk, consistency, directness, precision, and publication bias).^[10]^ And the quality of evidence will be rated as high, moderate, low, and very low.

## 3 Discussion

Colorectal polyp is a common disease of digestive tract, which can be divided into non-neoplastic and neoplastic. Non-neoplastic polyps mainly include inflammatory polyps, proliferative polyps, and juvenile polyps. The etiology of colorectal polyp mostly comes from gene mutation, gene suppression, and family inheritance. For example, familial adenomatous polyposis and Gardner syndrome are caused by gene mutation; the pathogenic gene of Peutz-Jeghers syndrome may be a tumor suppressor gene; Turcot syndrome and juvenile polyp are familial genetic diseases. Neoplastic polyps mainly refer to adenomatous polyps, with a high probability of canceration, which is the most important precancerous disease of colorectal cancer and has a rising trend in recent years.^[11]^

Clinical studies have shown that long-term use of natural or synthetic drugs, such as NSAIDs, may prevent polyps from developing in the colon, this effect known as chemoprophylaxis.^[12,13]^ Studies have confirmed that NSAIDs have a very significant protective effect on intestinal polyps, colon cancer, and rectal cancer, which can reduce the risk of intestinal polyps by 78%, colon cancer by 87%, and rectal cancer by 85%. However, smoking and drinking are also risk factors, whereas eating bean products and vegetables are protective factors. Therefore, when using such drugs to prevent the above diseases, we should pay attention to personal life and behavior habits. If we can cooperate with abstinence, quit smoking, and eat bean products and vegetables properly, we should also pay attention to the protection factors. In vitro studies also showed that NSAIDs and cyclooxygenase-2 (COX-2) inhibitors could inhibit the formation of colorectal tumors by inhibiting cell proliferation, inducing apoptosis, inducing cell cycle arrest, and interfering with tumor angiogenesis through cox-dependent and nondependent mechanisms.^[14,15]^

Sulindac is a precursor drug with minimal activity, which can be metabolized into active sulfide after entering the human body. It can inhibit cyclooxygenase and reduce the synthesis of prostaglandins, thus has analgesic, anti-inflammatory, and antipyretic effects. As early as 1983, Waddell and Loughry^[16]^ reported that sulindac had a regression effect on FAP patients. Researchers have found that the effect of sulindac in FAP patients act after 3 months. Polyps were significantly subsided in all patients after taking sulindac for 12 months, regardless of whether they had undergone surgery.^[17]^ Long-term maintenance therapy with sulindac can reduce the number of residual colorectal adenomas, the degree of heteromorphism and the proportion of villous tubular adenoma in patients, which may delay or prevent the occurrence of colon cancer. Piazza found that sulindac derivatives did not promote the differentiation of adenoma cells during cell culture, and induction of apoptosis was one of the main mechanisms. It was further speculated that sulindac may reduce the degree of adenoma atypia by inducing apoptosis of adenoma cells.^[18]^ So far, although sulindac is not enough to replace surgery, patients still need to regularly review colonoscopy, but it can be used as a beneficial supplement to surgical treatment.^[19]^

However, there is no systematic review and meta-analysis assessing the efficacy and safety of sulindac for colorectal polyps. This is the first protocol for systematic review and meta-analysis evaluating the efficacy and safety of sulindac for colorectal polyps. This systematic evaluation and meta-analysis can provide evidence-based evidence for clinicians to use sulindac in the treatment of colorectal polyps. However, the study has some limitations. Due to different dose and course of sulindac for colorectal polyps, the results were affected and the bias was caused. In addition, we only search for articles in Chinese and English, which may cause certain publication bias.

## Author contributions

**Data collection:** Qing Long and Liang Ao.

**Data curation:** Qing Long, Liang Ao.

**Formal analysis:** Qing Long.

**Funding acquisition:** Yan Li.

**Funding support:** Yan Li.

**Literature retrieval:** Liang Ao and Kuo Li.

**Resources:** Liang Ao, Kuo Li.

**Software:** Liang Ao.

**Supervision:** Yan Li.

**Writing – original draft:** Qing Long and Liang Ao.

**Writing – review & editing:** Qing Long and Yan Li.

## Abbreviations

Abbreviations: CBM = China biomedical literature database, CNKI = China National Knowledge Infrastructure, COX-2 = cyclooxygenase-2., FAP = familial adenomatous polyposis, GRADE = Grading of Recommendations Assessment, Development, and Evaluation, NSAIDs = nonsteroidal anti-inflammatory drugs, OSF = open science framework, PRISMA-P = preferred reporting items for systematic reviews and meta-analyses protocols, RCTs = randomized controlled trials, WHO = World Health Organization.
